# Editorial: Obligate intracellular bacteria: Evasion and adaptative tactics shaping the host-pathogen interface

**DOI:** 10.3389/fcimb.2022.965554

**Published:** 2022-07-14

**Authors:** Isaura Simões, Daniel E. Voth, Luís Jaime Mota

**Affiliations:** ^1^ CNC - Center for Neuroscience and Cell Biology, University of Coimbra, Coimbra, Portugal; ^2^ III - Institute of Interdisciplinary Research, University of Coimbra, Coimbra, Portugal; ^3^ Department of Microbiology and Immunology, University of Arkansas for Medical Sciences, Little Rock, AR, United States; ^4^ Associate Laboratory i4HB - Institute for Health and Bioeconomy, NOVA School of Science and Technology, NOVA University Lisbon, Caparica, Portugal; ^5^ UCIBIO – Applied Molecular Biosciences Unit, Department of Life Sciences, NOVA School of Science and Technology, NOVA University Lisbon, Caparica, Portugal

**Keywords:** obligate intracellular bacteria, host-pathogen interaction, evasion and control strategies, bacterial effector function, virulence mechanisms

Obligate intracellular bacteria are an important and fascinating group of microorganisms, as they are often pathogenic to humans and cause a significant clinical and public health burden worldwide. Adaptation to the obligate intracellular lifestyle implies an intimate and complex co-evolution with eukaryotic hosts over hundreds of millions of years. During this adaptation, these pathogens evolved sophisticated virulence mechanisms enabling them to grow intracellularly and resist host defences while minimizing damage to their hosts. This obligate intracellular nature is also a major research challenge, as these bacteria are typically not easy to handle and manipulate in the laboratory. However, over the last 15 years, several developments have significantly advanced understanding of the biology and virulence mechanisms of obligate intracellular bacterial pathogens. Some of these advances are covered in this Frontiers Research Topic.

Two reviews cover experimental models used to study *Chlamydia trachomatis* pathogenesis and evaluation of vaccines against Q fever (caused by *Coxiella burnetii*). Filardo et al. review the current knowledge on how *C. trachomatis* interacts with human prostate epithelial, Sertoli, and synovial cells and how studying *Chlamydia* survival and inflammatory host cell responses to infection in these cells aids elucidation of etiopathogenesis of *Chlamydia*-mediated male infertility, prostatitis, and reactive arthritis. On the importance of animal models as invaluable tools in preclinical evaluation of Q fever vaccine candidates and post-vaccination hypersensitivity modeling, Tesfamariam et al. thoroughly review available species and their unique advantages and limitations. Consideration of non-mammalian models in vaccine development efforts are also addressed, including the need to include gender and age as important factors when choosing a model to study disease pathogenesis and vaccine efficacy.

Many important insights can be obtained from the genomic analysis of obligate intracellular bacterial pathogens. Marti et al. review the knowledge on lateral gene transfer in the *Chlamydiaceae* and how intraspecies DNA exchange occurs more frequently than previously anticipated, even crossing species. This review also highlights the impact of homologous recombination on the chlamydial genome and current knowledge of chlamydial recombination machinery, while asking open questions about the function of recombinant-associated proteins. Moreover, the authors explore homologous recombination as a potential genetic tool in *Chlamydia*.

In another example, Verhoeve et al., use bioinformatics and phylogenomics analysis to identify and characterize a novel toxin-antidote module, composed of *REIS_1424* and *REIS_1423* genes that are uniquely expressed in *Rickettsia buchneri*. This novel rCRCT/CRCA module adds to a short list of factors that might explain the mutualism between *R. buchneri* and *Ixodes scapularis* and contribute to blocking superinfection of the blacklegged tick by other *Rickettsia*. This work opens exciting avenues of research on interbacterial warfare and the role of this endosymbiont in the biology of *Ixodes scapularis*, an important vector for several infectious disease agents.

Many bacterial pathogens use specialized secretion systems to deliver effector proteins into their host cells (Galán and Waksman, 2018). Three research articles highlight advances in the understanding of effectors’ functions.

*Anaplasma phagocytophilum* Ankyrin A (AnkA) is an important effector targeting the host cell nucleus that modifies the epigenome to promote bacterial fitness and propagation. Kim et al. investigate the AnkA nuclear import mechanism by generating mutations in N-terminal Ankyrin Repeats (ARs) to address the role of the RaDAR pathway combined with specific inhibition of the classical importin α/β pathway. Their results point toward the primacy of the importin-α/β system in AnkA nuclear localization, suggesting a supplemental or minor role for the RaDAR mechanism.

Aranjuez et al. utilize *Drosophila melanogaster* as a platform to study the impact of a *C. trachomatis* effector (Tarp) that has been associated with actin polymerization during chlamydial invasion. Here, the authors provide *in vivo* evidence that Tarp displays F-actin bundling activity and outcompetes the endogenous host bundler Fascin. This raises exciting hypotheses regarding multiple potential functions of Tarp in promoting *Chlamydia* invasion.

*Ehrlichia chaffeensis* is the causative agent of human monocytic ehrlichiosis, an emerging tick-borne infectious disease. *E. chaffeensis* replication within monocytes and macrophages relies on multiple proteins. Rikihisa focuses on recent findings related to the role of EtpE-C on invasion, functions of *E. chaffeensis* effectors Etf-1, -2, and -3 to facilitate intracellular replication, and *Ehrlichia* hijacking of host membrane lipids.

Another key aspect of the virulence of obligate intracellular bacteria is their ability to control host cell death. Wang and Cull highlight the current knowledge, challenges, and future perspectives regarding involvement of tick programmed cell death machinery (apoptosis and autophagy pathways) in tick-pathogen interactions. A deeper understanding of how these mechanisms and their interplay impact pathogen acquisition, replication, and transmission will ultimately identify novel approaches to controlling tick-borne diseases.

Finally, two research papers explore the importance of cellular O_2_ levels and how this is perceived and explored by pathogens. To dissect the impact of O_2_ concentration on growth of *Chlamydia*, Thapa et al. investigate the role of host NADPH oxidases and functional mitochondria in chlamydial growth under normoxia and hypoxia. Interestingly, their data show that *C. trachomatis* require functional mitochondria and NADPH oxidase 4/p38 MAPK signaling for growth under normoxia, opening interesting hypotheses about how *Chlamydia* might switch their energy source depending on cellular O_2_ concentration.

HIF1α is an important regulator of cellular responses to hypoxia and regulates transcription of genes involved in immune responses, metabolic reprogramming, and anti-infective responses. Hayek et al., provide evidence that *C. burnetii* infection destabilizes HIF1α and alters expression of multiple HIF1α target genes. The *C. burnetti* effector(s) responsible for this destabilization and mechanistic consequences for disease outcome remain interesting open questions.

In conclusion, this Research Topic provides a diverse range of topics on host-pathogen interactions, ranging from experimental models, genomics and evolution, protein effectors and their functions, control of host programmed cell death, and the impact of O_2_ concentration on infection. We thank all authors who contributed their work and all reviewers for their time and insightful comments that led to this exciting collection of articles.

## Author contributions

All authors listed have made a substantial, direct, and intellectual contribution to the work and approved it for publication.

## Funding

Work in IS laboratory was funded by the European Regional Development Fund (ERDF), through the COMPETE 2020 - Operational Programme for Competitiveness and Internationalisation and Portuguese national funds via FCT – Fundação para a Ciência e a Tecnologia, under project[s] POCI-01-0145-FEDER-029592 (PTDC/SAU-INF/29592/2017) (UNDOHIJACK), UIDB/04539/2020, and UIDP/04539/2020 (CIBB). Work in LJM laboratory has been supported by FCT through grants PTDC/BIA-MIC/28503/2017 (to LJM), UIDP/04378/2020 and UIDB/04378/2020 (UCIBIO), and LA/P/0140/2020 (i4HB).

## Conflict of Interest

The authors declare that the research was conducted in the absence of any commercial or financial relationships that could be construed as a potential conflict of interest.

## Publisher’s note

All claims expressed in this article are solely those of the authors and do not necessarily represent those of their affiliated organizations, or those of the publisher, the editors and the reviewers. Any product that may be evaluated in this article, or claim that may be made by its manufacturer, is not guaranteed or endorsed by the publisher.
